# Supplementary material for: Salvia Spp. Essential Oils against the Arboviruses Vector Aedes albopictus (Diptera: Culicidae): Bioactivity, Composition, and Sensorial Profile—Stage 1
Source: Biology (Basel). 2020 Aug 4;9(8):206. doi: 10.3390/biology9080206 (PMC7463507; doi:10.3390/biology9080206)
Supplement: Supplementary file 1 [file biology-09-00206-s001.pdf]

Supplementary materials

TableS1

Main botanical characteristics, propagation and cultivation methods, plants main uses, and essential oils (EOs) main uses of *Salvia dorisiana*, *S. dolomitica*, *S. sclarea,* and *S. somaliensis*.

Species	Botanical characteristics	Propagation and cultivation methods	Main uses	EO uses
***Salvia dolomitica* Codd** 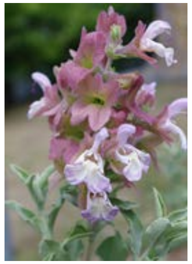	▪ branched shrub about 1-2 m tall<br>▪ Native of South Africa (North-East province of Transvaal)<br>▪ grow on heavy soils with dolomitic rocks (Ebani et al., 2018).<br>▪ Blooming in early spring with pink-white flowers in racemes.<br>▪ swelling calyxes<br>▪ leaves with balsamic fragrance (Cervelli, 2011)	▪ propagation by cutting.<br>▪ Plant growth fast.<br>▪ Water needs moderate.<br>▪ Grown in nurseries in pots	▪ Ornamental purposes (Bassolino et al., 2015).	▪ antiplasmodial and anti-inflammatory activities (Kamatou 2010, 2008, 2007).<br>▪ antimicrobial activity against bacteria, molds and yeasts (Ebani et al., 2018).<br>▪ anticancer properties against MCF7 a breast cancer cell line (IC50=37.05 µg/ml). (Kamotou et al., 2010)
***Salvia dorisiana* Standl.** 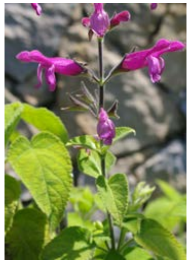	▪ shrub 1-1,5 m tall<br>▪ native of Central America (especially Honduras)<br>▪ big hearth-shaped light-green leaves<br>▪ large magenta-pink flowers<br>▪ Blooming from winter to spring.<br>▪ leaves with fruited fragrance (Cervelli, 2011)	▪ propagation by cutting.<br>▪ Plant growth fast.<br>▪ Water needs high.<br>▪ Grown in nurseries in pots<br>▪	▪ ornamental plant (Halim and Collins, 1975).<br>▪ as flavourings in salads and salad dressings and to make a flavourful tea (Facciola, 1998).	▪ The EO has been reported as the mosquito larvicidal activity and repellent effect (Conti et al., 2012b).

***Salvia somalensis* Vatke** 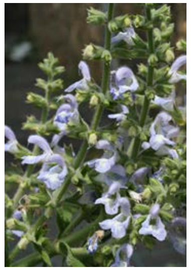	▪ branched shrub about 1-2 m tall<br>▪ native of the high elevation forest lands of Somalia<br>▪ Elliptic light-green leaves<br>▪ Blooming in spring with azure flowers in long inflorescens<br>▪ Fragrance lighter than Salvia officinalis (Cervelli, 2011)	▪ propagation by cutting.<br>▪ Plant growth moderate.<br>▪ Water needs low Grown in nurseries	▪ ornamental plant.	▪ antibacterial and antifungal (Ebani et al., 2018).
***Salvia sclarea* L.** 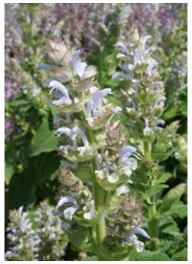	▪ herbaceous biennial or perennial<br>▪ native to Southern Europe<br>▪ ovate or hearth-shaped big leaves<br>▪ Blooming in late spring on long inflorescences 40-70 cm tall<br>▪ Moscate-like fragrance (Cervelli, 2011)	▪ propagation by seed.<br>▪ Plant growth fast.<br>▪ Water needs moderate.<br>▪ Flowering in the second year.<br>▪ Grown in nurseries in pots for garden decoration.	▪ Widely grown in temperate climates for extraction of "absolute", largely used in perfumery as fixative, whose main important compound is sclareol, an oxygenated diterpene (Arctander, 2019). Is also the basic compound for chemical synthesis of substitutes of ambergris.<br>▪ used for tobacco aromatization.<br>▪ Dried leaves and inflorescences are used for liqueur preparation.<br>▪ ornamental plant.	▪ Antifungal and antioxidant activity (Singh et al., 2019)<br>▪ used in perfumery industries as a source of fragrance with refreshing and long-lasting note (Aćimović et al., 2018).<br>▪ used in stress, tension, depression, insomnia, etc. (Yaseen et al., 2014). against gingivitis, stomatitis and aphthae (Kostić et al., 2017).<br>▪ analgesic, anti-inflamatory, antimicrobial and cytotoxic effects (Gulcin et al., 2004).
